# Baseline Data of Reproductive System in the JPHC Study

**DOI:** 10.2188/jea.11.6sup_75

**Published:** 2007-11-30

**Authors:** Shaw Watanabe, Yasuhiko Iwase, Yuriko Kikuchi

**Keywords:** reproductive history, menarche, menopause, prospective cohort study

## Abstract

Hormonal status in the body is closely related to the occurrence of estrogen-related cancers. Baseline survey data about the female reproductive system in JPHC study showed different gynecological and gestational profiles in each study area. Late menarche (15-16 y/o) was characteristic in the rural areas. Earlier gestational age and larger number of children were also more common in the rural areas. Baseline survey data, including gynecological past history, frequency of examination for uterine cancers, and so forth, showed some profile of middle aged women in the different areas in Japan.

## INTRODUCTION

Japanese show the low incidence of breast cancer, endometrial cancer, and ovarian cancer compared to the Caucasians. Duration and amount of estrogen level is considered to be a risk factor for these cancers^1^^)^. Baseline survey of the JPHC study partially clarified the factors which being related to the geographic differences of the above described cancers in the female reproductive system.

## METHODS

Information about the female reproductive system was obtained from the female session of two kinds of self-administered baseline questionnaires. Questions about age of menarche, age of menopause, regularity and days of menstruation cycle, any history of hormone therapy, number of pregnancy and children, age of gestation and childbirth, breast feeding, participation to the uterine and/or breast mass screening program, and past history of gynecological diseases were asked.

Several questions in the questionnaires were different in Cohort I and II. In terms of hormone treatment, “Have you ever used female sex hormones in the present or past?” in Cohort I was revised to “Have you ever used female sex hormones for contraception, dysmenorrhea or climacteric distress?” in Cohort II. Gynecological past history was selected from the list of diseases, such as “none, mastopathy, mastitis, mammary tumor, endometritis, uterine myoma, ovarian cyst, vaginitis, and others” in Cohort I, but vaginitis was deleted in Cohort II. The question about mass screening for uterine and mammary cancers was moved to the question for both sexes about an experience to participate to the mass-screening program performed by the local government, such as stomach, lung, colon, uterus and breast in Cohort II.

## RESULTS

Age of cohort subjects was from 40 to 59 years in Cohort I and from 40 to 69 years in Cohort II. It may cause systematic bias about age of menarche and number of gestation and children due to the rapid change of society after the World War II.

Average age of menarche was 16.4 year and 15.0 year in both area of Okinawa, such as Miyako and Ishikawa, respectively. It was 13.2 years in Suita and 13.4 years in Katsushika (Table 1). In other areas, it was averagely 14 years. Age of menarche in metropolitan areas was earlier than other rural areas, and it was 2-3 years older in Okinawa. On the contrary, age of menopause did not show such clear trend, but almost 2 years difference was present in Cohort I (47.8 years) and Cohort II (49.4 years) (Table 2).

**Table 1.** Age disribution of menarche.

													(%)

Age (yr)	Ninohe	Yokote	Saku	Ishikawa	Katsusihka	Kasama	Kashiwazaki	Tosayamada	Arikawa	Miyako	Suita 1	Suita 2	Total

<10	0.5	0.3	0.3	0.1	1.5	0.3	0.5	0.6	0.3	0.1	0.9	0.6	0.4
11	1.7	0.9	1.3	0.7	5.0	1.4	1.8	3.3	1.2	0.6	5.5	3.7	2.0
12	9.1	8.6	8.5	4.3	20.9	9.4	10.2	16.5	9.1	3.5	22.8	16.7	10.8
13	19.8	17.5	16.8	14.2	28.3	21.8	15.7	25.5	17.9	9.7	31.2	26.7	20.0
14	26.5	23.6	24.3	23.6	25.7	30.2	28.0	27.3	30.5	17.6	25.8	25.7	25.7
15	21.2	25.1	22.4	23.7	13.5	22.3	24.7	16.0	23.4	21.4	10.2	15.8	20.6
16	10.9	11.7	11.6	14.2	3.2	8.5	8.1	6.4	9.4	14.0	2.3	5.8	9.5
17	5.8	5.9	6.6	9.6	1.0	3.1	5.1	2.8	4.0	10.9	1.0	2.9	5.1
18	2.8	4.1	4.6	5.4	0.6	2.0	4.0	1.3	2.8	10.7	0.3	1.2	3.4
19	0.9	1.3	2.8	2.6	0.2	0.6	1.3	0.2	0.7	6.6	0.1	0.5	1.5
>20	0.7	0.9	0.8	1.5	0.1	0.4	0.6	0.2	0.7	4.9	0.0	0.5	0.9

Number of subjects	4651	6034	5207	5235	3933	6653	1000	2642	3819	3341	3563	1541	47619
Mean	14.4	14.6	14.6	15.0	13.4	14.2	14.4	13.7	14.3	15.7	13.2	13.7	14.3
S.D.	1.7	1.7	1.8	1.8	1.4	1.5	1.7	1.5	1.6	2.1	1.3	1.6	1.8


**Table 2.** Age distribution of natural menopause.

													(%)

Age (yr)	Ninohe	Yokote	Saku	Ishikawa	Katsusihka	Kasama	Kashiwazaki	Tosayamada	Arikawa	Miyako	Suita 1	Suita 2	Total

-29	0.1	0.0	0.0	0.0	0.0	0.0	0.0	0.0	0.1	0.0	0.4	0.0	0.0
30-34	0.2	0.1	0.2	0.3	0.3	0.1	0.2	0.0	0.2	0.1	0.2	0.0	0.2
35-39	1.0	0.9	1.1	1.5	2.2	0.7	0.4	0.8	0.8	1.4	1.1	0.1	1.0
40-44	6.0	6.4	5.8	5.5	4.4	4.9	4.4	4.9	6.1	6.3	5.7	3.6	5.6
45-49	41.7	38.9	35.7	37.9	75.4	37.2	35.9	32.9	36.6	39.5	85.6	30.7	40.6
50-54	48.1	50.4	54.1	52.0	17.8	54.2	54.2	58.0	52.7	49.5	7.0	60.8	49.5
55-59	2.9	3.3	3.1	2.9	0.0	2.9	4.8	3.4	3.5	3.3	0.0	4.8	3.0

Number of subjects	2006	2913	2423	2184	780	2318	454	759	1647	1462	557	691	18194
Mean	49.0	49.1	49.3	49.2	47.8	49.4	49.7	49.7	49.3	49.0	47.6	50.0	49.2
S.D.	3.4	3.4	3.5	3.5	2.5	3.2	3.1	3.1	3.5	3.5	2.7	3.0	3.4


Menstruation cycle was regular in about 80% women in all districts (Table 3a, b). Average menstruation cycle was 28 days.

**Table 3a.** Menstrual cycle.

		Ninohe		Yokote		Saku		Ishikawa		Katsusihka		Total	

	Days	n	%	n	%	n	%	n	%	n	%	n	%
	<19	44	1.4	38	1.0	14	0.4	12	0.3	1	0.0	109	0.6
	20-21	43	1.4	34	0.9	42	1.3	77	1.8	31	1.1	227	1.3
	22-23	40	1.3	67	1.7	59	1.9	66	1.5	47	1.6	279	1.6
	24-25	321	10.1	440	11.4	387	12.1	705	16.3	395	13.5	2248	12.9
	26-27	218	6.9	305	7.9	238	7.5	478	11.1	235	8.0	1474	8.4
	28-29	1863	58.8	2069	53.7	1739	54.6	2039	47.2	1438	49.2	9148	52.4
	30-31	583	18.4	836	21.7	661	20.7	895	20.7	676	23.1	3651	20.9
	32-33	20	0.6	16	0.4	20	0.6	18	0.4	33	1.1	107	0.6
	34-35	20	0.6	33	0.9	26	0.8	23	0.5	39	1.3	141	0.8
	>36	17	0.5	14	0.4	1	0.0	4	0.1	27	0.9	63	0.4

Total		3169		3852		3187		4317		2922		17447	
Mean		27.6		27.8		27.7		27.6		28.0		27.7	
S.D.		3.4		3.2		2.6		2.4		2.4		2.8	
No answer		292		655		540		107		122		1716	

**Table 3b.** Menstrual cycle.

	Kasama		Kashiwazaki		Tosayamada		Arikawa		Miyako		Suita 1		Suita 2		Total	

Days	n	%	n	%	n	%	n	%	n	%	n	%	n	%	n	%
<25	690	10.2	107	12.7	248	11.3	335	7.0	520	12.2	411	15.3	193	10.0	2504	10.7
26	220	3.2	27	3.2	58	2.6	87	1.8	86	2.0	135	5.0	64	3.3	677	2.9
27	201	3.0	23	2.7	74	3.4	163	3.4	140	3.3	107	4.0	58	3.0	766	3.3
28	3057	45.0	408	48.3	1136	51.8	2307	48.4	1822	42.6	1235	46.1	994	51.6	10959	46.7
29	138	2.0	25	3.0	50	2.3	46	1.0	38	0.9	73	2.7	52	2.7	422	1.8
30	2289	33.7	240	28.4	550	25.1	1765	37.0	1576	36.9	600	22.4	465	24.1	7485	31.9
>31	193	2.8	15	1.8	75	3.4	66	1.4	90	2.1	118	4.4	102	5.3	659	2.8

Total	6788		845		2191		4769		4272		2679		1928		23472	
Mean	28.4		28.1		28.3		28.5		28.3		28.0		28.4		28.3	
S.D.	2.3		2.4		2.8		1.8		2.1		2.3		2.3		2.2	
No answer	297		396		477		40		170		117		14		1511	

About 18% women used any hormonal drug in the past or present in Cohort I, but it was less than 10% in Cohort II (Table 4a, b). Number of gestation was 3.1 (2.7-3.9) in Cohort I, and 3.2 (2.6-4.4) in Cohort II. It was high in rural areas such as Miyako and Arikawa, and low in metropolitan city (Table 5). Number of delivery was 0.5 smaller than that of pregnancy. Age of first delivery was younger in Tohoku (23 years of age), and it was 25 years in Katsushika, Suita and Saku (Table 6). Average number of delivery was 2.6 (Table 7). Number of children was 1-2 in 46.7% and 3-4 in 39.3%. More than 7-8 children were reported in Arikawa (4.3%), Ishikawa (5.3%) and Miyako (3.7%), while the other areas were less than 1%. Age of first childbirth was 20-24 in 45.8% women and 25-29 in 42.4% women, in average (Table 8). In general, more than half women in rural areas tended to deliver at younger age, while it was reverse in large city, such as Katsushika (Tokyo) and Suita. Saku showed the urban pattern, although it belonged primarily to agricultural district. Proportion of women who had delivered after age 40 years was also high in both districts of Okinawa, Ishikawa and Miyako.

**Table 4a.** Take of hormone drug.

													(n)

Hormone therapy	Ninohe	Yokote	Saku	Ishikawa	Katsusihka	Kasama	Kashiwazaki	Tosayamada	Arikawa	Miyako	Suita 1	Suita 2	Total

None	3537	4656	4026	4173	3246	8621	1476	3422	5504	3932	3136	2150	47879
Past	995	1090	919	933	606	491	44	313	220	156	351	160	6278
Now	84	92	92	114	59	43	4	48	18	51	27	20	652

Total	4616	5838.	5037	5220	3911	9155	1524	3783	5742	4139	3514	2330	54809
No answer	257	445	440	77	73	404	121	213	474	1130	68	59	3761

	4873	6283	5477	5297	3984	9559	1645	3996	6216	5269	3582	2389	58570


**Table 4b.** Take of hormone drug.

														(%)

Hormone therapy		Ninohe	Yokote	Saku	Ishikawa	Katsusihka	Kasama	Kashiwazaki	Tosayamada	Arikawa	Miyako	Suita 1	Suita 2	Total

None		76.6	79.8	79.9	79.9	83.0	94.2	96.9	90.5	95.9	95.0	89.2	92.3	87.4
Past		21.6	18.7	18.2	17.9	15.5	5.4	2.9	8.3	3.8	3.8	10.0	6.9	11.5
Now		1.8	1.6	1.8	2.2	1.5	0.5	0.3	1.3	0.3	1.2	0.8	0.9	1.2

Total	(n)	4616	5838	5037	5220	3911	9155	1524	3783	5742	4139	3514	2330	54809
No answer	(n)	257	445	440	77	73	404	121	213	474	1130	68	59	3761


**Table 5.** Number of pregnancy.

													(%)

Pregnancy n	Ninohe	Yokote	Saku	Ishikawa	Katsusihka	Kasama	Kashiwazaki	Tosayamada	Arikawa	Miyako	Suita 1	Suita 2	Total

0	5.1	5.1	4.8	7.4	6.8	4.8	3.3	7.3	6.5	4.5	8.9	8.6	6.0
1-2	31.6	41.7	28.7	14.9	38.8	46.6	33.9	4.2	17.5	13.9	40.8	36.0	32.7
3-4	45.9	44.6	54.2	40.1	42.4	42.3	53.9	41.5	44.8	41.9	40.2	41.1	44.2
5-6	14.3	7.6	11.3	27.6	10.8	5.9	8.4	8.0	24.2	30.4	8.9	12.6	13.9
7-8	2.8	0.7	0.9	7.9	1.1	0.4	0.5	0.9	5.6	7.7	1.0	1.4	2.6
9-10	0.3	0.2	0.1	1.8	0.1	0.0	0.0	0.2	1.2	1.4	0.2	0.3	0.5
11-	0.1	0.0	0.0	0.3	0.0	0.0	0.0	0.1	0.3	0.2	0.1	0.1	0.1

Number of subjects	4606	5787	5043	5232	3885	6292	946	2552	3501	2629	3513	1520	45506
Mean	3.1	2.7	3.0	3.9	2.8	2.6	2.9	2.7	3.7	4.1	2.6	2.8	3.1
S.D.	1.6	1.3	1.4	2.1	1.5	1.2	1.2	1.4	1.9	1.9	1.5	1.6	1.7


**Table 6.** Age of first pregnancy.

													(%)

Pregnant age (yr)	Ninohe	Yokote	Saku	Ishikawa	Katsusihka	Kasama	Kashiwazaki	Tosayamada	Arikawa	Miyako	Suita 1	Suita 2	Total

<19	10.0	3.0	1.3	9.5	3.9	2.7	1.8	6.9	6.1	5.3	2.6	2.2	4.8
20-24	59.9	64.0	40.3	47.9	44.7	52.4	62.5	61.5	58.7	55.3	47.2	47.2	52.9
25-29	25.6	29.2	51.4	30.5	41.1	39.1	31.9	26.0	28.7	31.6	42.0	43.0	35.3
30-34	3.6	2.8	5.8	9.0	7.8	4.7	3.3	4.6	5.1	5.6	6.4	6.4	5.5
35-39	0.9	0.9	1.1	2.5	2.4	1.0	0.3	0.9	1.2	1.8	1.7	0.9	1.4
>40	0.1	0.0	0.1	0.5	0.2	0.1	0.2	0.2	0.2	0.2	0.1	0.2	0.2

Number of subjects	4305	5407	4744	4836	3590	5910	901	2399	3236	2443	3169	1383	42263
Mean	23.3	23.8	25.3	24.4	25.1	24.5	24.1	23.6	23.9	24.1	25.0	24.9	24.3
S.D.	3.4	2.9	2.9	4.3	3.7	3.0	2.8	3.4	3.5	3.7	3.4	3.2	3.5

* Only women who had ever experienced pregnancy

**Table 7.** Number of child birth.

													(%)

Children n	Ninohe	Yokote	Saku	Ishikawa	Katsusihka	Kasama	Kashiwazaki	Tosayamada	Arikawa	Miyako	Suita 1	Suita 2	Total

0	5.2	4.9	4.8	7.6	8.6	5.3	3.7	7.7	6.9	4.9	10.7	10.4	6.5
1-2	42.8	60.4	41.8	20.2	59.9	57.5	46.4	65.6	25.2	19.6	63.7	61.6	46.7
3-4	45.3	33.1	50.4	46.8	30.4	35.5	47.3	25.6	48.5	52.1	24.7	27.2	39.3
5-6	5.9	1.4	2.8	20.1	1.0	1.6	2.4	0.9	15.0	19.7	0.8	0.7	6.2
7-8	0.7	0.1	0.2	4.2	0.1	0.0	0.2	0.1	3.6	3.4	0.1	0.0	1.1
9-10	0.1	0.1	0.0	1.0	0.0	0.0	0.0	0.0	0.6	0.3	0.0	0.0	0.2
11-	0.0	0.0	0.0	0.1	0.0	0.0	0.0	0.0	0.1	0.0	0.0	0.0	0.0

Number of subjects	4630	5826	5095	5187	3913	6402	956	2615	3628	2761	3512	1523	46048
Mean	2.6	2.3	2.6	3.5	2.1	2.3	2.5	2.1	3.2	3.5	2.0	2.0	2.6
S.D.	1.3	1.0	1.1	1.9	1.0	1.0	1.0	1.0	1.7	1.6	1.0	1.0	1.4


**Table 8.** Age of first child birth.

													(%)

Birth age (yr)	Ninohe	Yokote	Saku	Ishikawa	Katsusihka	Kasama	Kashiwazaki	Tosayamada	Arikawa	Miyako	Suita 1	Suita 2	Total

<19	5.7	1.2	0.6	6.0	1.2	1.2	0.6	3.2	2.5	2.4	0.8	1.0	2.4
20-24	56.4	55.3	30.8	44.5	34.8	43.8	52.4	57.7	53.4	51.2	36.2	37.4	45.8
25-29	31.8	38.0	58.5	34.9	49.8	46.7	42.2	31.9	36.0	36.8	51.2	50.0	42.4
30-34	4.8	4.0	8.6	10.9	10.9	6.7	4.1	5.9	6.2	6.8	9.3	9.9	7.4
35-39	1.3	1.4	1.4	3.0	3.1	1.5	0.6	1.2	1.6	2.2	2.3	1.4	1.8
>40	0.1	0.1	0.1	0.6	0.2	0.2	0.1	0.2	0.3	0.5	0.2	0.3	0.3

Number of subjects	4313	5437	4790	4777	3559	5944	898	2376	3290	2556	3119	1346	42405
Mean	23.9	24.6	26.0	25.1	26.0	25.2	24.7	24.2	24.6	24.8	25.9	25.7	25.1
S.D.	3.5	3.0	3.0	4.4	3.6	3.1	2.8	3.4	3.5	3.8	3.3	3.2	3.5


Breast-feeding was reported by 81.6-93.9% mother (Table 9a, b). It exceeded 90% in Kashiwazaki, Arikawa and Miyako in Cohort II.

**Table 9a.** Proportion of breast feeding in Cohort I .

Breast feeding	Ninohe		Yokote		Saku		Ishikawa		Katsusihka		Total	

	n	%	n	%	n	%	n	%	n	%	n	%
Yes	3787	87.4	4524	82.6	4196	87.5	4131	86.5	2903	81.6	19541	85.2
No	544	12.6	953	17.4	600	12.5	647	13.5	655	18.4	3399	14.8

Total	4331		5477		4796		4778		3558		22940	
No answer	58		63		53		14		19		207	

**Table 9b.** Proportion of breast feeding in Cohort II .

Breast feeding	Kasama		Kashiwazaki		Tosayamada		Arikawa		Miyako		Suita 1		Suita 2		Total	

	n	%	n	%	n	%	n	%	n	%	n	%	n	%	n	%
Yes	7167	85.3	1341	91.9	2895	83.6	4882	93.1	3636	93.9	2658	85.6	1762	86.6	24341	88.3
No	1232	14.7	118	8.1	568	16.4	362	6.9	236	6.1	448	14.4	272	13.4	3236	11.7

Total	8399		1459		3463		5244		3872		3106		2034		27577	
No answer	87		19		66		95		122		31		33		453	

Proportion of mass-screening examinees within one year was 37.8-56.3% for uterine cancer, and 17.6-54.2% for breast cancer (Table 10a, b).

**Table 10a.** Participant rate to uteine cancer screening in Cohort I .

Mass screening	Ninohe		Yokote		Saku		Ishikawa		Katsusihka		Total	

	n	%	n	%	n	%	n	%	n	%	n	%
Yes	2039	42.7	3011	48.1	2891	56.3	2414	46.1	1490	37.8	11845	46.7
No	2731	57.3	3245	51.9	2248	43.7	2827	53.9	2452	62.2	13503	53.3

Total	4770		6256		5139		5241		3942		25348	
No answer	103		27		338		56		42		566	

**Table 10b.** Participant rate to uteine cancer screening in Cohort II .

Mass screening	Ninohe		Yokote		Saku		Ishikawa		Katsusihka		Total	

	n	%	n	%	n	%	n	%	n	%	n	%
Yes	1766	37.1	2048	32.8	2773	54.2	1870	35.7	691	17.6	9148	36.2
No	2992	62.9	4197	67.2	2340	45.8	3367	64.3	3244	82.4	16140	63.8

Total	4758		6245		5113		5237		3935		25288	
No answer	115		38		364		60		49		626	

Gynecological past history was not present in 62-77% women, 70.7% in Cohort I and 65.2% in Cohort II in average (Table 11a, b). Uterine myoma, mastitis, vaginitis, mastopathy, and ovarian cyst were common diseases in the past history. The frequency was different by cohort areas. Diagnosis of uterine myoma was more frequent in Cohort II, especially in Tosayamada (18%) and Suita (17%). Vaginitis was reported from about 5% women in Saku and Katsushika. Past history of mammary tumor was less than 1% except for Suita.

**Table 11a.** Past gynecological history in Cohort I .

Past history	Ninohe		Yokote		Saku		Ishikawa		Katsusihka		Total	

	n	%	n	%	n	%	n	%	n	%	n	%
None	3739	76.7	4427	70.5	3435	62.7	4087	77.2	2641	66.3	18329	70.7
Mastopathy	130	2.7	282	4.5	369	6.7	76	1.4	116	2.9	973	3.8
Mastitis	221	4.5	429	6.8	466	8.5	251	4.7	320	8	1687	6.5
Mammary gland tumor	24	0.5	28	0.4	32	0.6	17	0.3	27	0.7	128	0.5
Endometritis	535	11	881	14	954	17.4	496	9.4	602	15.1	3468	13.4
Uterine myoma	103	2.1	136	2.2	204	3.7	152	2.9	111	2.8	706	2.7
Ovarian cystoma	104	2.1	157	2.5	162	3	97	1.8	138	3.5	658	2.5
Vaginitis	146	3	239	3.8	280	5.1	185	3.5	226	5.7	1076	4.2
Others	81	1.7	105	1.7	120	2.2	134	2.5	133	3.3	573	2.2

Total	4873		6283		5477		5297		3984		25914	
No answer	246		96		398		57		39		836	

**Table 11b.** Past gynecological history in Cohort II .

Past history	Kasama		Kashiwazaki		Tosayamada		Arikawa		Miyako		Suita 1		Suita 2		Total	

	n	%	n	%	n	%	n	%	n	%	n	%	n	%	n	%
None	6508	68.1	1145	69.6	2193	54.9	4168	67.1	3523	66.9	2273	63.5	1481	62	21291	65.2
Mastopathy	363	3.8	41	2.5	555	13.9	76	1.2	66	1.3	201	5.6	92	3.9	1394	4.3
Mastitis	537	5.6	113	6.9	350	8.8	352	5.7	186	3.5	301	8.4	241	10.1	2080	6.4
Mammary gland tumor	88	0.9	17	1	33	0.8	30	0.5	12	0.2	46	1.3	37	1.5	263	0.8
Endometritis	235	2.5	14	0.9	99	2.5	166	2.7	88	1.7	162	4.5	90	3.8	854	2.6
Uterine myoma	1205	12.6	169	10.3	722	18.1	764	12.3	270	5.1	573	16	413	17.3	4116	12.6
Ovarian cystoma	340	3.6	37	2.2	141	3.5	126	2	110	2.1	139	3.9	94	3.9	987	3
Others	192	2	36	2.2	94	2.4	138	2.2	71	1.3	131	3.7	107	4.5	769	2.4

Total	9559		1645		3996		6216		5269		3582		2389		32656	
No answer	568		125		204		612		1036		45		51		2641	

## DISCUSSION

Active duration of estrogen secretion is closely related to the occurrence of breast, endometrial, and ovarian cancer^1^^-^^3^^)^. Higher age of menarche among Japanese was considered to result in low incidence of estrogen-related cancers. Recent studies suggested that the dietary interaction like phytoestrogens modifies the effect of estrogen to reduce the risk of cancer and other gynecological distress^4^^-^^6^^)^.

Proportion of many answers by women in Cohort I and II was different. The age of subjects in Cohort I was between age 40 and 59, while that in Cohort II was from age 40 to 69. This 10-year difference in age 60s reflected the dramatic change of lifestyle among Japanese around the World War II. Number of gestation, pregnancy, and children had changed after the war. Age of menarche and first pregnancy was different in rural and urban areas. Saku is known as an area of high education, so attitude of women seemed to be similar to those in urban city.

Mortality of uterine cervical cancer was steadly dropped since 1960. After the mass screening program was installed in 1983, high screening rate could decrease the mortality rate to 5/10,000 by early treatment^7^^)^. However, the participation rates to mass screening test was less than half in most areas. The education for hygiene and improvement of housing condition had contributed more to decrease the incidence rate. Rate of endometritis, vaginitis and uterine myoma in the past history varied much by area. The chance to go to gynecologists may be a factor to cause such difference, because the rural areas showed relatively high proportion to have had above diseases in the past history. Mammary tumor also showed the same trend, and only Suita subjects reported the tumor in more than 1%.

Cancer registration system has been made in each cohort area and works in high quality, so the risk and preventive factors could be more clearly shown based upon the histologically verified cases. Extension of the epidemiological study to osteoporosis and climacteric distress may be also possible in the JPHC Cohort in the future, if the proper diagnosis could be collected at the time of follow-up study.
